# Early Life Predictors of Increased Body Mass Index among Indigenous Australian Children

**DOI:** 10.1371/journal.pone.0130039

**Published:** 2015-06-15

**Authors:** Katherine A. Thurber, Timothy Dobbins, Martyn Kirk, Phyll Dance, Cathy Banwell

**Affiliations:** 1 National Centre for Epidemiology and Population Health, Research School of Population Health, The Australian National University, Canberra, Australia; 2 ANU Medical School, The Australian National University, Canberra, Australia; 3 National Centre for Indigenous Studies, The Australian National University, Canberra, Australia; Swinburne University of Technology, AUSTRALIA

## Abstract

Aboriginal and Torres Strait Islander Australians are more likely than non-Indigenous Australians to be obese and experience chronic disease in adulthood—conditions linked to being overweight in childhood. Birthweight and prenatal exposures are associated with increased Body Mass Index (BMI) in other populations, but the relationship is unclear for Indigenous children. The Longitudinal Study of Indigenous Children is an ongoing cohort study of up to 1,759 children across Australia. We used a multilevel model to examine the association between children’s birthweight and BMI z-score in 2011, at age 3-9 years, adjusted for sociodemographic and maternal factors. Complete data were available for 682 of the 1,264 children participating in the 2011 survey; we repeated the analyses in the full sample with BMI recorded (n=1,152) after multilevel multiple imputation. One in ten children were born large for gestational age, and 17% were born small for gestational age. Increasing birthweight predicted increasing BMI; a 1-unit increase in birthweight z-score was associated with a 0.22-unit (95% CI:0.13, 0.31) increase in childhood BMI z-score. Maternal smoking during pregnancy was associated with a significant increase (0.25; 95% CI:0.05, 0.45) in BMI z-score. The multiple imputation analysis indicated that our findings were not distorted by biases in the missing data. High birthweight may be a risk indicator for overweight and obesity among Indigenous children. National targets to reduce the incidence of low birthweight which measure progress by an increase in the population’s average birthweight may be ignoring a significant health risk; both ends of the spectrum must be considered. Interventions to improve maternal health during pregnancy are the first step to decreasing the prevalence of high BMI among the next generation of Indigenous children.

## Introduction

Aboriginal and Torres Strait Islander Australians experience severe health inequities; their average life expectancy is around a decade shorter than that of non-Indigenous people [1]. Two-thirds of this disparity has been attributed to the burden of chronic diseases [2]. Attempts to reduce this gap and the burden of chronic disease have thus far been unsuccessful. Better and earlier prevention strategies are required [3]. Birthweight, as well as prenatal and early postnatal influences, may place infants on a trajectory to develop chronic disease in adulthood. It is critical to understand these early influences to improve health outcomes.

A strong link exists between childhood weight status and the development of chronic diseases in adulthood [4]. Preventing the gain of excess weight in childhood presents an opportunity to intervene and prevent the development of chronic diseases. Aboriginal and Torres Strait Islander people are significantly more likely to be obese than non-Indigenous people at almost every age, including childhood [5], and the gap may be widening [6]. In 2012–13, 6.0% of Aboriginal and Torres Strait Islander children aged 2–4 years and 11.2% of those 5–9 years were obese, compared to 5.1% and 7.6% of non-Indigenous children in the respective age groups in 2011–12 [5].

Examination of the link between birthweight and childhood weight ‘has immediate relevance’ [3p. 1662] for the Aboriginal and Torres Strait Islander population, given the elevated rates of low birthweight [7], obesity, and chronic diseases [2, 5]. This link has been explored within other populations [8], but current evidence about the association, particularly concerning high birthweight, for Aboriginal and Torres Strait Islander Australians is inconclusive [3, 9] with most research focused on the impacts of low birthweight. Around 18,000 Aboriginal and Torres Strait Islander children are born in Australia annually [10]. Given the different cultural context, setting, maternal health profile, and health burden, it is necessary to confirm this relationship within Aboriginal and Torres Strait Islander children in order to inform improvements to child health.

To better understand the association between prenatal exposures, birthweight—across the whole spectrum—and childhood Body Mass Index (BMI), we examined data from the Longitudinal Study of Indigenous Children (LSIC), a prospective national cohort study of Aboriginal and Torres Strait Islander children in Australia. We hypothesised that birthweight z-score would predict BMI z-score, and that an independent association would persist after adjustment for potential confounders.

## Materials and Methods

### Study Population: the Longitudinal Study of Indigenous Children (LSIC)

**Fig 1:**
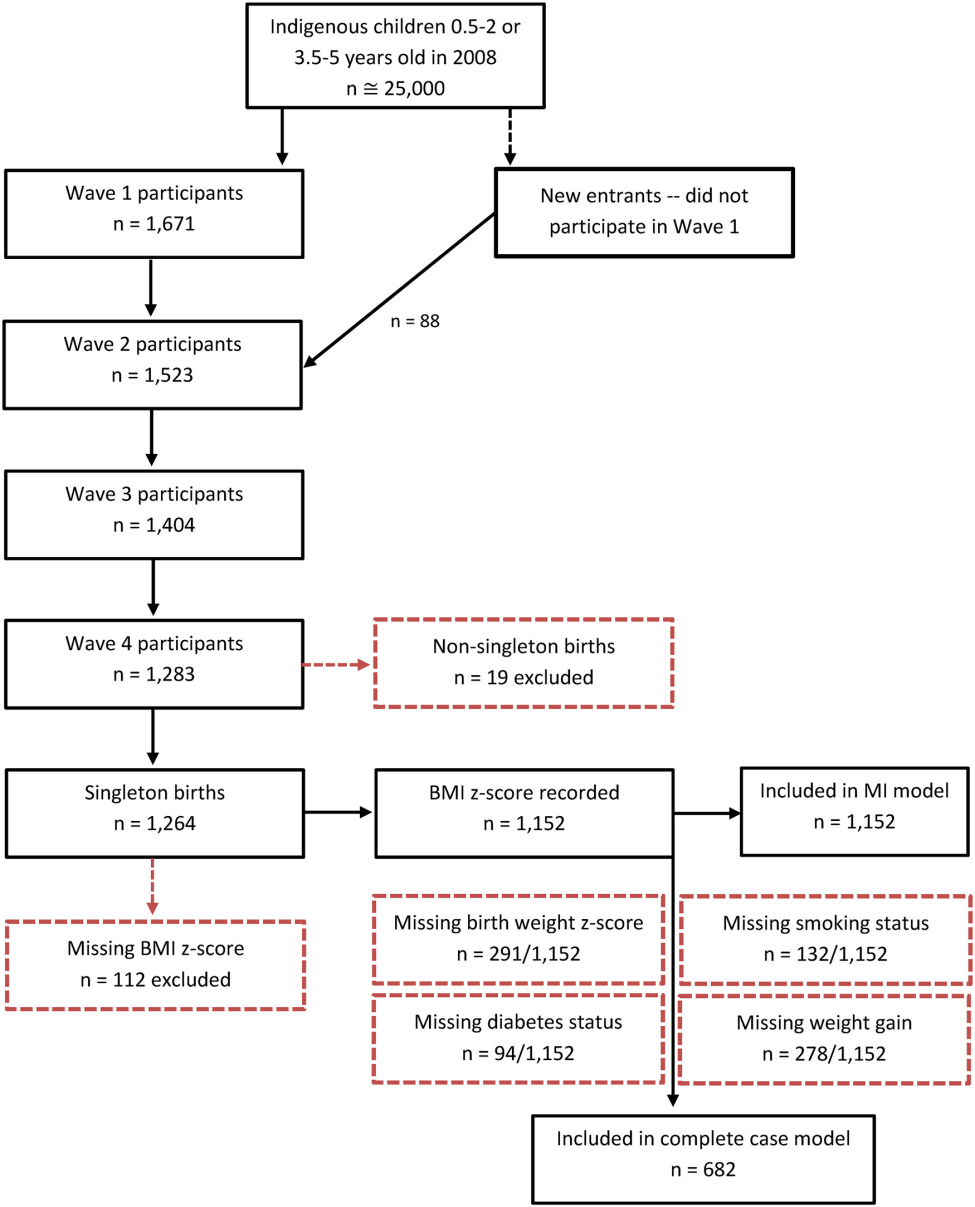
Flow chart of the LSIC study population. * These numbers refer to interviews with the primary carer.

LSIC is a national longitudinal study aimed to increase understanding about the development and wellbeing of Aboriginal and Torres Strait Islander children. The study is managed by the Australian Government Department of Social Services (DSS). At the time of this study (2013), LSIC had collected four waves of data on up to 1,759 Aboriginal and Torres Strait Islander children across Australia, representing 5–10% of the population of that age (Fig 1). Purposive sampling was used to recruit children from 11 diverse sites; the sampling design, and its implications for analyses, has been described elsewhere [11, 12]. In this study, we examined data from 1,264 children participating in Wave 4, collected in 2011.

### Data and variables

#### Indigenous Area

For protection of privacy, LSIC does not release data on the site from which children were sampled, but they provide randomised codes for the Indigenous Area (IARE) in which each child resides. IARE is an Australian Bureau of Statistics measure of spatial location (comparable to the Statistical Area 3 for the overall Australian population) [13], and each Area represents a smaller geographic unit than the 11 non-disclosed sampling sites. This measure allows for children living in the same geographic area to be grouped together and has been demonstrated to effectively account for the study’s clustered sampling design [12].

#### Outcome variable

LSIC interviewers measured the weight and height of children at each wave of the study. At Wave 4, Homedics model SC-305-AOU-4209 digital scales were used to weigh children, and Soehnle professional Model 5003 stadiometers were used to measure their standing height. If parents and carers were uncomfortable having their children weighed or measured, the most recent measurements were taken from their child’s health record book (0.5% of measurements in Wave 4). To improve the validity of the data, a cleaning method based on WHO standards and protocols was developed and employed [14]. Plausible measurements were recorded for 91.1% of the sample (n = 1,152/1,264).

Metric measurements were used to calculate each child’s BMI (kilograms/metres^2^). We calculated BMI z-scores, specific to age and sex, using the World Health Organization international reference [15, 16]. We used continuous BMI z-score as the primary outcome because an increase in BMI z-score, even within the normal range, is associated with increased risk of chronic disease in adulthood [17, 18]. Where appropriate, we have used BMI z-scores to categorise children as underweight, normal weight, overweight, or obese based on the age-specific BMI cut-off points [16]. These BMI cut-off points were derived to link to the corresponding BMI cut-offs in adults [19]. More conservative cut-off points are used to define overweight and obesity for children ≤5 years of age, compared to those 5–18 years of age, due to the potentially different implications of ‘excess’ weight for children undergoing different stages of growth [19]. Participants missing data on BMI z-score were excluded (n = 112/1,264) from analyses, leaving 1,152 children for this study.

#### Predictors

The child’s birth mother was asked to provide the child’s birth weight and gestational age from the child’s health book (‘Baby Book’) at the first Wave of the study. If mothers could not access the Baby Book, they were asked to report this information from memory (19% of recorded birth weights).

We calculated birthweight z-scores, adjusted for gestational age and sex, to disentangle the effects of gestational age and foetal growth rate [20, 21]. We used a national reference of Australian singleton births from 1998 to 2007 for standardisation [22]; thus our analyses were restricted to singleton births (excluding 19 children). The use of a continuous scale for birthweight, rather than categories based on arbitrary cut-offs, enables representation of the magnitude of the deviance from the expected birthweight. Birthweight z-scores have demonstrated improved prediction of BMI compared to raw birthweight, even when adjusted for gestational age [23]. Plausible birthweight z-scores were recorded for 74.7% of the sample with recorded BMI z-score (n = 861/1,152).

In the absence of a nationally agreed definition, cut-off points of z = -1.28 and z = +1.28 were used in alignment with defined percentile cut-offs, to categorise children as small for gestational age (SGA, falling in the lowest decile of births for gestational age and gender), appropriate for gestational age (AGA), and large for gestational age (LGA, falling in the highest decile).

#### Potential confounders

We included the child’s age group (categorised as 3–4, 4–5, 5–7, or 7–9 years), sex (male or female), and Indigenous identification (self-reported identification as Aboriginal, Torres Strait Islander, or both). Although Aboriginal and/or Torres Strait Islander people are often grouped together as ‘Indigenous’, they represent distinct groups and were considered separately.

We examined area-level socioeconomic status as well as maternal factors known to be associated with birthweight or childhood BMI through a physiological mechanism: smoking, diabetes, and weight gain during pregnancy. These factors were chosen to be consistent with other research in this area. The aim was not to be exhaustive, but to examine if the inclusion of these variables altered the association between birthweight and BMI.

Maternal smoking during pregnancy was determined based on mothers’ response to the question, ‘After finding out you were pregnant with (STUDY CHILD) did you smoke any cigarettes during the pregnancy?’ Mothers were recorded as having diabetes in pregnancy if they reported having ‘Diabetes or sugar problems’ or taking any ‘Sugar diabetes medication/insulin’ during the child’s pregnancy. Mothers were asked, ‘During your pregnancy, did your doctor or nurse tell you that your weight gain was too much, okay or not enough?’ Responses were categorised as ‘too much’ or ‘okay or not enough’ weight gain; data were coded as missing if the mother did not speak to a doctor or nurse about weight gain.

We measured current (Wave 4) area-level socioeconomic status using the Index of Relative Indigenous Socioeconomic Outcomes (IRISEO). IRISEO is based on nine measures of socio-economic status (including employment, education, income, and housing), and calculated specifically for Indigenous Australians [24]. The LSIC sample is relatively evenly distributed across the IRISEO population deciles. Three categories of area-level disadvantage were created for the LSIC sample: most advantaged (IRISEO 8–10), mid-advantaged (IRISEO 4–7), and most disadvantaged (IRISEO 1–3).

### Statistical analysis

A multilevel approach was employed to account for the survey’s clustered sampling design [12]. Although LSIC collects longitudinal data, we examined only the most recent measurement of BMI.

BMI z-score was the outcome variable, and birthweight z-score was the primary explanatory variable. Linearity of the association between birthweight z-score and childhood BMI z-score was assessed graphically, and a non-linear association was not indicated. The IARE was included as a random effect to account for the correlation structure inherent to the LSIC survey design.

Four models were constructed, with potential confounders added in steps; variables were retained in subsequent models regardless of significance as these were selected for inclusion *a priori*. The first model regressed BMI z-score on birthweight z-score, age category, sex, and Indigenous identification. The second model added the potential physiological confounders to Model 1, and the third model added area-level disadvantage to Model 1. The final model included all variables. The degree of clustering was summarised using the intraclass correlation coefficient (ICC).

With the exception of BMI, physiological measures were collected via self-report, and were subject to non-response. The final analysis was based on participants with complete data (n = 682). Exclusions and the final samples are displayed in Fig 1. As a secondary analysis, to assess potential biases arising from the complete case analysis, we used multilevel multiple imputation (using the program REALCOM-impute [25]) to conduct the same analyses in the sample providing data on BMI in the Wave 4 of LSIC (n = 1,152). Multiple imputation is a commonly used approach to help reduce bias or increase precision resulting from missing data in epidemiological and clinical research [26]. All other analyses were conducted using Stata version 12.1.

### Ethics Statement

The LSIC survey was conducted with ethical approval from the Departmental Ethics Committee of the Australian Commonwealth Department of Health, and from the Human Research Ethics Committees of each state and territory. The Australian National University’s Human Research Ethics Committee granted ethical approval for the current analysis of LSIC in October 2011 (Protocol No. 2011/510).

## Results

The mean BMI z-score at Wave 4 (2011) was 0.28 (95% CI:0.19, 0.36). The majority of children (79.3%) fell within the normal range for BMI, with 4.3% underweight, and 16.4% overweight or obese (Table 1). The mean birthweight z-score was -0.17 (95%CI:-0.25, -0.10); 16.5% of the sample was SGA and 10.6% LGA. Children were relatively evenly distributed across age groups; the vast majority (n = 1,027) identified as Aboriginal, with 71 identifying as Torres Strait Islander and 54 identifying as both. Around 50% of mothers reported smoking during pregnancy, 6.7% reported diabetes during pregnancy, and 12.2% reported too much weight gain. The majority lived in areas with mid-level advantage, with 18.7% in the most advantaged and 20.7% in the most disadvantaged areas.

**Table 1:** Distribution of Body Mass Index in LSIC Wave 4 (2011), across potential demographic and physiological confounders. ^a^

	n	Mean BMI z-score	95% CI	% Underweight	% Normal weight	% Overweight / obese
**Total**	1,152	0.28	[0.19, 0.36]	4.3	79.3	16.4
**Sex**						
Male	582	0.28	[0.16, 0.39]	4.5	78.7	16.8
Female	570	0.27	[0.16, 0.39]	4.2	79.8	16.0
**Age**						
3–4 years	237	0.36	[0.19, 0.54]	3.4	86.1	10.6
4–5 years	401	0.28	[0.14, 0.41]	4.2	87.0	8.7
5–7 years	245	0.20	[0.02, 0.37]	4.5	73.5	22.0
7–9 years	269	0.26	[0.08, 0.45]	5.2	66.9	27.9
**Indigenous identification**						
Aboriginal	1,027	0.27	[0.18, 0.35]	4.4	79.7	16.0
Torres Strait Islander	71	0.43	[0.08, 0.77]	5.6	73.2	21.1
Both	54	0.25	[-0.13, 0.62]	1.9	79.6	18.5
**Size for gestational age category**						
SGA	142	-0.04	[-0.27, 0.18]	5.6	81.0	13.4
AGA	628	0.45	[0.34, 0.55]	2.6	79.1	18.3
LGA	91	0.66	[0.36, 0.97]	4.4	73.6	22.0
Missing	291	-0.06	[-0.23, 0.11]	7.6	80.4	12.0
**Diabetes status during pregnancy**						
No diabetes	987	0.29	[0.21, 0.38]	4.0	80.5	15.6
Diabetes	71	0.64	[0.27, 1.02]	1.4	71.8	26.8
Missing	94	-0.16	[-0.51, 0.18]	10.6	72.3	17.0
**Smoking during pregnancy**						
No	513	0.26	[0.14, 0.38]	3.5	79.9	16.6
Yes	507	0.31	[0.19, 0.44]	4.5	79.5	16.0
Missing	132	0.18	[-0.10, 0.45]	6.8	75.8	17.4
**Weight gain during pregnancy**						
Okay or not enough	767	0.25	[0.15, 0.35]	4.8	79.4	15.8
Too much	107	0.77	[0.48, 1.05]	0.0	71.0	29.0
Missing	278	0.16	[-0.02, 0.33]	4.7	82.0	13.3
**Area-level advantage/disadvantage at Wave 1**						
Most advantaged	215	0.43	[0.23, 0.62]	3.7	78.6	17.7
Mid-advantaged	699	0.41	[0.31, 0.51]	3.0	78.4	18.6
Most disadvantaged	238	-0.27	[-0.45, -0.08]	8.8	82.4	8.8

^a^ Includes only the sample with no missing data on BMI z-score. BMI categories were defined based on WHO standard cut-offs, which are more conservative for children ≤5 years compared to >5 years of age [16, 19]. Size for gestational age categories were defined using cut-off points of z = -1.28 and z = +1.28 were used, in alignment with standard percentile cut-offs.

There was a non-significant trend towards decreasing mean BMI z-score with children’s increasing age, but the prevalence of combined overweight and obesity was higher in the older group. This apparent discrepancy is largely explained by the change in BMI cut-offs at age 5 years [16, 19]. In the younger age group, with more conservative cut-off points, 6.1% of children were overweight and 3.6% obese. Among children over 5 years of age, 13.6% were overweight and 11.4% obese.

The mean BMI z-score was significantly lower in the most disadvantaged, compared to more advantaged, areas, and significantly higher among children whose mothers reported gaining ‘too much’ weight during pregnancy, compared to ‘okay’ or ‘not enough’ weight. The mean BMI z-score was elevated for children whose mothers reported having diabetes or smoking during pregnancy compared to mothers who did not, but these differences were not significant in the bivariate analyses.

**Fig 2:**
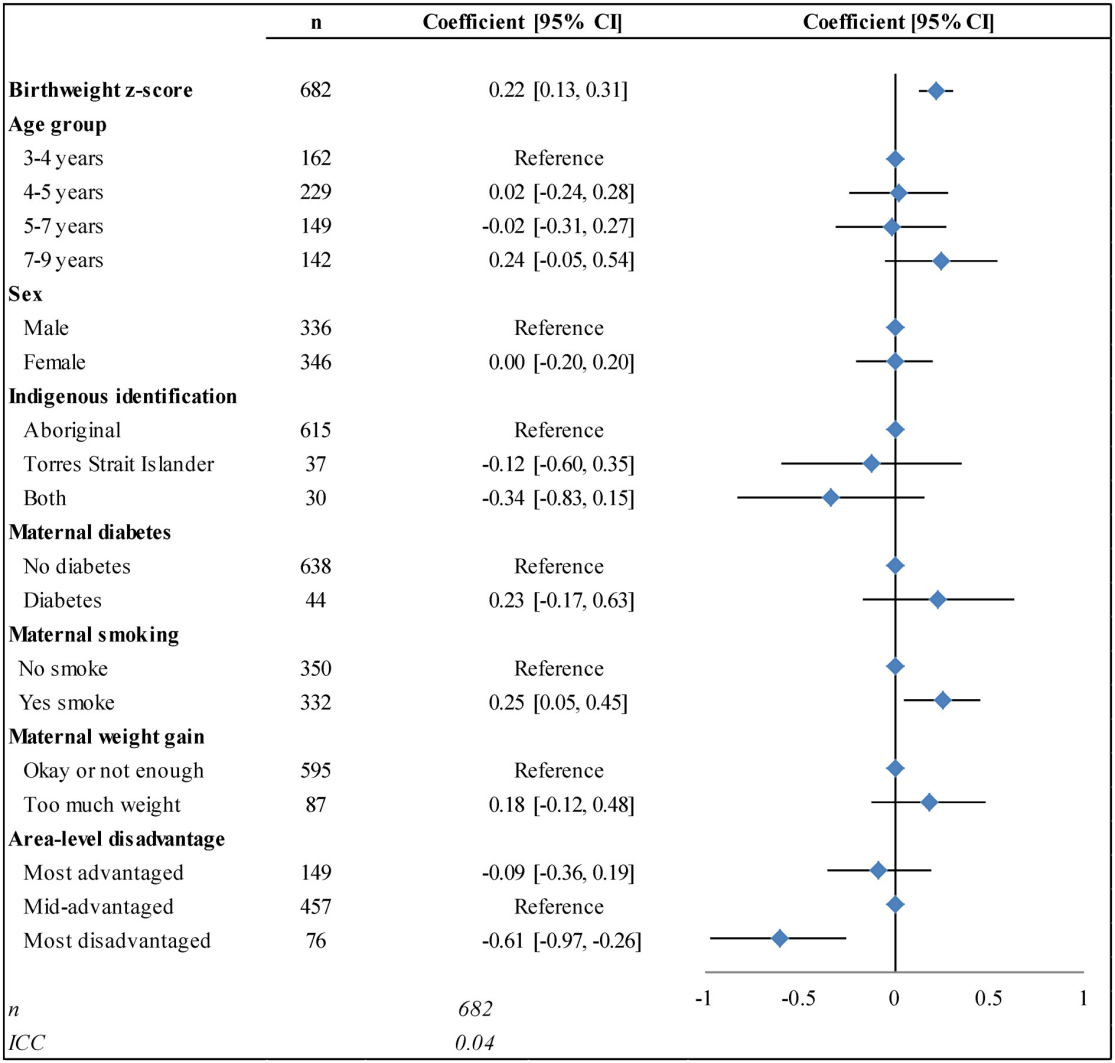
The association between BMI z-score, birthweight z-score, demographic factors, and physiological factors. *The final model was adjusted for: age group; Indigenous identification; maternal diabetes, smoking, and weight gain during pregnancy; and area-level socioeconomic status. See S1 Table for the results of the preliminary models.

In the final multilevel model, there was a significant positive linear association between birthweight z-score and BMI z-score (Fig 2). The inclusion of the full set of explanatory variables did not alter the coefficient for birthweight, and improved the fit of the model, although the sample size was reduced. Every 1-unit increase in birthweight z-score was associated with a 0.22-unit increase in BMI z-score (95% CI:0.13, 0.31). A child born LGA (with a birthweight z-score of +1.28) would be predicted to have a BMI z-score 0.28 units higher than a child born at the median, and 0.56 units higher than a child born SGA (with a birthweight z-score of -1.28).

Smoking during pregnancy was associated with a 0.25-unit increase (95% CI:0.05, 0.45) in BMI z-score in the full model, and living in a disadvantaged area was associated with a 0.61-unit decrease (95% CI:-0.97, -0.26). Age group, sex, and Indigenous identification were not significantly associated with BMI z-score. Reporting maternal diabetes or ‘too much’ weight gain during pregnancy was associated with a non-significant increase in BMI z-score (0.23; 95% CI:-0.17, 0.63 and 0.18; 95% CI:-0.12, 0.48, respectively).

We conducted a sensitivity analysis to compare the complete case analysis to analysis of the full dataset using multilevel multiple imputation (S1 File), and found relatively consistent results, suggesting that biases in the missing data did not distort our findings. While the effect of birthweight did not change substantially, the effect of maternal weight gain and diabetes was strengthened.

## Discussion

This study is the first to examine the relationship between BMI and the whole spectrum of birthweight among Aboriginal and Torres Strait Islander children. These results suggest that higher birthweight indicates an increased risk of obesity in childhood and therefore an increased risk of adult onset chronic disease. Due to the focus on low birthweight, high birthweight has not yet been considered a risk indicator for Aboriginal and Torres Strait Islander children. Further, this study identifies smoking during pregnancy as an independent risk factor for increased BMI in this sample.

Our study demonstrates a positive, linear association between birthweight z-score and BMI z-score through age 3–9 years in a sample of Aboriginal and Torres Strait Islander children. The association persisted after adjusting for demographic factors, a set of physiological factors, and area-level socioeconomic status. A 1-unit change in BMI z-score can represent a shift from normal weight to overweight, or from overweight to obese; thus, a 0.28-unit increase in BMI z-score attributable to being born LGA (compared to the median birthweight) is substantial. Further, research suggests that the risk of chronic disease increases with increasing childhood BMI z-score, even within the normal range [17, 18].

Nationally, there was a significant increase in the mean birthweight of singleton babies born to Aboriginal and Torres Strait Islander mothers from 2000–2011 [27]. In the LSIC sample, we also observed temporal trend towards increasing birthweight z-score from 2003–2008 (S2 Table). The continuation of this trend could have detrimental implications for the weight status of future generations of Aboriginal and Torres Strait Islander children.

Smoking during pregnancy was associated with significantly lower birthweight z-scores in this sample, consistent with other research (S2 Table). It was also significantly associated with increased BMI z-score in the final model, independent of birthweight. This is consistent with research in other populations, although the mechanism is not well understood [28]. However, this relationship has not been previously explored within the Aboriginal and Torres Strait Islander populations [29].

Half of the mothers in the LSIC sample reported smoking during their child’s pregnancy, consistent with national estimates (48%) for Aboriginal and Torres Strait Islander mothers in 2012 [30]. Around 6,000 infants born to Aboriginal and Torres Strait Islander mothers in 2012 were exposed to smoke in utero. Given the high rate of smoking during pregnancy for Aboriginal and Torres Strait Islander women—more than three times the rate for non-Indigenous women [30]—this may be a critical modifiable risk factor for childhood obesity in this population. Further, a dose-response relationship has been observed between the number of cigarettes smoked daily during pregnancy and the child’s risk of obesity [31], suggesting that any reduction in mothers’ smoking during pregnancy could have a positive health impact.

Compared to children living in the more advantaged areas, children living in disadvantaged areas had significantly lower BMI z-scores in childhood even after adjusting for birthweight. The effect was strengthened after adjusting for the physiological factors (S1 Table), indicating that the association was not a result of confounding due to these factors. The representation of children from all levels of advantage is a strength of LSIC, in comparison to other studies which are localised in nature, allowing for robust analysis.

In our study, maternal weight gain and diabetes were both non-significant predictors of increased childhood BMI z-score. This might suggest that these variables are not associated with childhood BMI in this sample independent of their impact on birthweight. However, the lack of significance for these relationships may be partially attributable to the small numbers (n = 87 and n = 44, respectively, in the final model; both coefficients were strengthened in the multiple imputation analysis), or to the lack of precision in these measures. Despite the lack of significance of these indicator variables in this study, maternal obesity and diabetes are likely to be important contributors to obesity among Aboriginal and Torres Strait Islander children, either independently [32], or through their association with increased birthweight (S2 Table) [33, 34]. This is of concern given the high rates of, and increasing trends in, both obesity and diabetes among Aboriginal and Torres Strait Islander women of reproductive age [35, 36].

These findings are consistent with a systematic review of prenatal exposures and later cardio-metabolic conditions in Indigenous populations from Australia, New Zealand, Canada, and the US [3]. In the two studies of Australian Indigenous populations, lower birthweight was associated with lower BMI in adults. Importantly, our study also examines the trajectory of children born at the other end of the birthweight spectrum, identifying higher birthweight as a risk indicator for obesity.

Our findings are also consistent with a global meta-analysis of studies in non-Indigenous populations [8], and findings from the Longitudinal Study of Australian Children, a nationally representative study of around 10,000 children, predominantly non-Indigenous. Children with high birthweight, compared to those with normal or low birthweight, faced an increased risk of obesity at age 4–5 years [37]. Thus, national and international data—for both Indigenous and non-Indigenous populations—consistently suggest an association between increasing birthweight and increasing childhood BMI.

These findings suggest that the first step in preventing obesity among the next generation of Aboriginal and Torres Strait Islander children is to improve the health of their mothers. Interventions should be targeted at the earliest possible stage: during or, optimally, before pregnancy. Pregnancy, a period marked by increased health care interaction, offers an ideal ‘window of opportunity’ to reduce the intergenerational transmission of chronic disease risk. Factors leading to both low and high birthweight—including maternal smoking, overweight, and diabetes in pregnancy—are often entrenched in disadvantage, and shaped by social and cultural norms. Thus, improving maternal health during pregnancy would require accessible, effective antenatal care, including social and psychological support [38].

There are potential limitations to our study. Although obesity is most accurately assessed by measuring percentage body fat [39], it is not feasible to measure this in most large-scale studies. BMI serves as a proxy measure, and its use has increasingly gained acceptance in public health research [40]. The reliance on self-reported data has the potential to induce bias. Recall bias in reported birthweight would likely underestimate the association between birthweight and BMI because mothers tend to overestimate birthweight for small infants, and underestimate for large infants [3]. Previous analyses of reported gestational age in this study suggest that the data should not unduly affect the calculation of z-scores [41].

Maternal healthcare information was not accessible, so we relied on mothers’ recall of their healthcare interactions during their pregnancy. In this study, we did not have a measure of maternal BMI during pregnancy, so we examined reported weight gain during pregnancy. The proportion of mothers reporting ‘too much’ weight gain, diabetes, or smoking during pregnancy likely underestimates the prevalence of maternal overweight/obesity, diabetes and smoking in the sample. Thus, any bias would more likely underestimate the strength of the observed associations. The small sample number of mothers reporting gestational diabetes or ‘too much’ weight gain during pregnancy prevented meaningful exploration of the interplay between risk factors with opposing influences on birthweight (e.g. mothers reporting both smoking and diabetes in pregnancy). The risk of bias due to residual confounding resulting from the limited inclusion of potential confounders should be minimal; a systematic review of the relationship between birthweight and risk of overweight indicated that the lack of inclusion of even ‘potentially critical’ confounders such as gestational age had no significant impact on the overall results [8].

LSIC is not nationally representative, although this national study does offer diversity in geography and environmental conditions. As is common among cohort studies, the LSIC data are designed for internal comparisons and longitudinal analyses [42]. The subset of children with BMI and birthweight z-scores recorded may not fully represent the entire LSIC sample; however, the findings of the multiple imputation analysis (S1 File) suggest that biases in the missing data did not distort our findings.

## Conclusions

Although many studies have now identified an association between high birthweight and later risk of obesity, the policy focus for Aboriginal and Torres Strait Islander Australians has remained on low birthweight, which has demonstrated detrimental impacts on health. Policies and targets to ‘improve’ birthweight aim to reduce the prevalence of low birthweight, often measuring improvement by an increase in the average birthweight of the population [7]. Given that an increase in average birthweight could arise either from a decreased prevalence of low birthweight or from an increased prevalence of high birthweight, this may not be an accurate indicator of the population’s health. Efforts are required to reduce the prevalence of high birthweight, alongside efforts to reduce the prevalence of low birthweight, and to improve the health of mothers during pregnancy.

## Supporting Information

S1 File Multilevel multiple imputation. (DOCX)

S1 Table Results of the models with potential confounders added in steps. (DOCX)

S2 Table Distribution of birthweight among children participating in Wave 4 of LSIC (2011), across demographic and physiological variables. (DOCX)
