# Supplementary material for: Early Life Predictors of Increased Body Mass Index among Indigenous Australian Children
Source: PLoS One. 2015 Jun 15;10(6):e0130039. doi: 10.1371/journal.pone.0130039 (PMC4468174; doi:10.1371/journal.pone.0130039)
Supplement: S1 File — (DOCX) [file pone.0130039.s001.docx]

**S1 File**. **Multilevel multiple imputation**

Analysis models need to take account of a study’s design for results to be unbiased. This is also true for multiple imputation models. Using single-level multiple imputation methods for a clustered survey with unbalanced data (such as LSIC) could result in biased parameter estimates and invalid estimates of precision [[1](#_ENREF_1), [2](#_ENREF_2)].

The multiple imputation methods available in Stata 12.1 do not allow for a multilevel structure; the REALCOM-IMPUTE program was developed to fill this gap [[1](#_ENREF_1), [2](#_ENREF_2)]. This program is freely available from <http://www.cmm.bristol.ac.uk/research/Realcom/index.shtml>, and code to export data between REALCOM-IMPUTE and Stata is available from <http://www.missingdata.org.uk/>. REALCOM-IMPUTE fits the model using Markov Chain Monte Carlo and a Gibbs sampling approach.

Before creating an imputation model, we explored variables related to the values and missingness of each variable in the analysis model. We focused on birthweight z-score as it had the most missing data of variables in the analysis model [[3](#_ENREF_3)], and its missingness was related to the missingness of three other variables (maternal diabetes, smoking, and weight gain during pregnancy). Missingness of birthweight z-score was not related to missingness of BMI z-score; however, it was significantly associated with the value of BMI z-score. Missingness of birthweight z-score was also significantly associated with age group and area-level disadvantage, factors included in the analysis model.

Remoteness, measured using the Level of Relative Isolation (LORI) scale, was significantly related to the value of BMI z-score, and the missingness of both BMI and birthweight z-scores. Thus, this variable was chosen for inclusion in the multiple imputation model to help uphold the missing at random assumption [[1](#_ENREF_1), [3](#_ENREF_3), [4](#_ENREF_4)].

First, we fit our analysis model in Stata using complete case analysis. Next, we exported the data to REALCOM-IMPUTE, identifying the variables to be imputed (“response variables”), the level two variables, and the auxiliary variables (covariates with no missing data). Response variables were identified as either continuous, ordinal, or unordered categorical.

The multiple imputation model included all variables in the analysis model (including the outcome variable, BMI z-score) and the categorical variable indicating remoteness. We included BMI z-score and birthweight z-score as continuous variables (each with an approximately normal distribution); and maternal diabetes, smoking, and weight gain as unordered categorical variables to be imputed. Children missing BMI z-scores were included in the imputation model as they provided additional information on the relationships between the exposures. We did not analyse the imputed BMI z-scores, however, to avoid adding noise to the estimates [[5](#_ENREF_5)]; the imputation model was restricted to children with non-missing BMI data only (n=1,152).

Variables with no missing data – sex, age category, Indigenous identification, disadvantage, and remoteness – were included as auxiliary variables. Dummy variables were created for categorical auxiliary variables. Disadvantage deciles were collapsed into quintiles to avoid small cell sizes. The Indigenous Area variable was included as the level-2 identifier. We included all other variables as level-1 variables as in the analysis model.

Following suggestions from Carpenter et al, we chose a burn-in period of 500 iterations, creating imputations at every 500th iteration [[2](#_ENREF_2)]. We chose to compute 50 imputations (for a total of 25,000 iterations), in line with common guidelines [[5](#_ENREF_5), [6](#_ENREF_6)].

The imputations were exported back into Stata, and we checked that the distribution of imputed variables resembled the original distribution in the sample. The distribution of variables across the total LSIC sample (with BMI z-score recorded), the sample included in the complete case analysis, the imputed data, and the full sample (with BMI z-score recorded) including imputed values is presented in **S1.1 Table**.

**S1.1 Table.** **Distribution of variables in the full sample, complete case analysis, and imputed datasets.**

|  | **# missing** | **Sample w/ BMI**  **(n = 1,152 - # missing)** | **Complete cases**  **(n = 682)** | **Imputed values***  **(n = # missing)** | **Imputed + original**  **(n = 1,152)** |
| --- | --- | --- | --- | --- | --- |
|  |  |  |  |  |  |
| Mean BMI z-score [95% CI] | -- | 0.28  [0.19, 0.36] | 0.37  [0.27, 0.47] | -- | 0.28  [0.19, 0.36] |
| Mean birthweight z-score [95% CI] | 291 | -0.17  [-0.25, -0.10] | -0.19  [-0.27, -0.10] | -0.17  [-0.42, 0.07] | -0.17  [-0.25, -0.09] |
|  |  |  |  |  |  |
|  |  | **Proportion** | **Proportion** | **Proportion** | **Proportion** |
|  |  |  |  |  |  |
| **Age group** | 0 |  |  |  |  |
| 3-4 years |  | 20.6 | 23.8 | -- | 20.6 |
| 4-5 years |  | 34.8 | 33.6 | -- | 34.8 |
| 5-7 years |  | 21.3 | 21.9 | -- | 21.3 |
| 7-9 years |  | 23.4 | 20.8 | -- | 23.4 |
|  |  |  |  |  |  |
| **Sex** | 0 |  |  |  |  |
| Male |  | 50.5 | 49.3 | -- | 50.5 |
| Female |  | 49.5 | 50.7 | -- | 49.5 |
|  |  |  |  |  |  |
| **Indigenous identification** | 0 |  |  |  |  |
| Aboriginal |  | 89.2 | 90.2 | -- | 89.1 |
| Torres Strait Islander |  | 6.2 | 5.4 | -- | 6.2 |
| Both |  | 4.7 | 4.4 | -- | 4.7 |
| **Maternal diabetes** | 94 |  |  |  |  |
| No diabetes |  | 93.3 | 93.6 | 91.7 | 93.2 |
| Yes diabetes |  | 6.7 | 6.5 | 8.3 | 6.8 |
| **Maternal smoking** | 132 |  |  |  |  |
| No smoke |  | 50.3 | 51.3 | 48.0 | 50.0 |
| Yes smoke |  | 49.7 | 48.7 | 52.0 | 50.0 |
| **Maternal weight** **gain** | 278 |  |  |  |  |
| Okay or not enough |  | 87.8 | 87.2 | 89.5 | 88.2 |
| Too much |  | 12.2 | 12.8 | 10.5 | 11.8 |
| **Area-level disadvantage** | 0 |  |  |  |  |
| Most advantaged |  | 18.7 | 21.9 | -- | 18.7 |
| Middle advantage |  | 60.7 | 67.0 | -- | 60.7 |
| Most disadvantaged |  | 20.7 | 11.1 | -- | 20.7 |
| **Level of relative isolation** | 0 |  |  |  |  |
| No |  | 28.7 | 34.0 | -- | 28.7 |
| Low |  | 47.1 | 51.0 | -- | 47.0 |
| Moderate |  | 14.8 | 10.0 | -- | 14.8 |
| High/extreme |  | 9.4 | 5.0 | -- | 9.4 |

* These data represent the proportion estimation across the 50 imputed datasets.

The analysis model was re-fit for each imputed data set, combined using Rubin's rules (**S1.2 Table**). The results of the two methods were very similar, with relatively consistent coefficients and p-values.

**S1.2 Table. Results of the complete case analysis and multiple imputation analysis.**

|  | **Complete case analysis** (Model 4) | **Multiple imputation analysis** (Model 4 MI) |
| --- | --- | --- |
|  | **Coefficient [95% CI]** | **Coefficient [95% CI]** |
| **Birthweight z-score** | 0.22 [0.13, 0.31] | 0.19 [0.11, 0.27] |
| **Age group** | | |
| 3-4 years | Reference | Reference |
| 4-5 years | 0.02 [-0.24, 0.28] | -0.03 [-0.25, 0.19] |
| 5-7 years | -0.02 [-0.31, 0.27] | -0.14 [-0.38, 0.10] |
| 7-9 years | 0.24 [-0.05, 0.54] | -0.02 [-0.26, 0.22] |
| **Sex** | | |
| Male | Reference | Reference |
| Female | 0.00 [-0.20, 0.20] | 0.01 [-0.14, 0.17] |
| **Indigenous identification** | | |
| Aboriginal | Reference | Reference |
| Torres Strait Islander | -0.12 [-0.60, 0.35] | -0.10 [-0.49, 0.29] |
| Both | -0.34 [-0.83, 0.15] | -0.14 [-0.53, 0.24] |
| **Maternal diabetes** | | |
| No diabetes | Reference | Reference |
| Diabetes | 0.23 [-0.17, 0.63] | 0.29 [-0.04, 0.62] |
| **Maternal smoking** | | |
| No smoke | Reference | Reference |
| Yes smoke | 0.25 [0.05, 0.45] | 0.17 [0.00, 0.35] |
| **Maternal weight gain** | | |
| Okay or not enough | Reference | Reference |
| Too much weight | 0.18 [-0.12, 0.48] | 0.36 [0.07, 0.64] |
| **Area-level disadvantage** | | |
| Most advantaged | -0.09 [-0.36, 0.19] | 0.03 [-0.24, 0.30] |
| Mid-advantaged | Reference | Reference |
| Most disadvantaged | -0.61 [-0.97, -0.26] | -0.69 [-0.97, -0.40] |
| N | 682 | 1,152 |
| ICC | 0.04 | 0.08 |

In the multiple imputation analysis, the magnitude of the coefficient for ‘too much’ weight gain during pregnancy increased from 0.18 to 0.36, crossing the threshold for significance (at α = 0.05). This variable had the highest prevalence of missing data, at n=278/1,152. The proportion of the sample with mothers gaining ‘too much’ weight gain was very similar in the original sample and in the imputed data (12.2% versus 10.5%).

The coefficient for smoking during pregnancy decreased from 0.25 to 0.17, but maintained significance at α = 0.05, with p=0.047. The coefficient for maternal diabetes during pregnancy increased from 0.23 to 0.29, with an associated decrease in p-value from 0.262 to 0.083.

References

1. Goldstein H, Carpenter J, Kenward MG, Levin KA. Multilevel models with multivariate mixed response types. Statistical Modelling. 2009;9(3):173-97.

2. Carpenter JR, Goldstein H, Kenward MG. REALCOM-IMPUTE software for multilevel multiple imputation with mixed response types. J Stat Softw. 2011;45(5):1-14.

3. Spratt M, Carpenter J, Sterne JA, Carlin JB, Heron J, Henderson J, et al. Strategies for multiple imputation in longitudinal studies. Am J Epidemiol. 2010;172(4):478-87.

4. Sterne JA, White IR, Carlin JB, Spratt M, Royston P, Kenward MG, et al. Multiple imputation for missing data in epidemiological and clinical research: potential and pitfalls. BMJ. 2009;338:b2393.

5. White IR, Royston P, Wood AM. Multiple imputation using chained equations: issues and guidance for practice. Stat Med. 2011;30(4):377-99.

6. Social Science Computing Cooperative. Multiple Imputation in Stata: Introduction Madison: University of Wisconsin; 2013 [updated 31 July 2014, accessed 01 August 2014]. Available from: <http://www.ssc.wisc.edu/sscc/pubs/stata_mi_intro.htm>.
