# Supplementary material for: Early Life Predictors of Increased Body Mass Index among Indigenous Australian Children
Source: PLoS One. 2015 Jun 15;10(6):e0130039. doi: 10.1371/journal.pone.0130039 (PMC4468174; doi:10.1371/journal.pone.0130039)
Supplement: S1 Table — (DOCX) [file pone.0130039.s002.docx]

**S1 Table.** **Results of the models with potential confounders added in steps.**

|  | ***Model 1:**  Demographic variables | **^†^Model 2:**  Demographic and physiological variables | **^‡^Model 3:**  Demographic variables and area-level SES | **^§^Model 4:**  Full model |
| --- | --- | --- | --- | --- |
|  | **Coefficient**  **[95% CI]** | **Coefficient**  **[95% CI]** | **Coefficient**  **[95% CI]** | **Coefficient**  **[95% CI]** |
| **Birthweight z-score** | 0.20 [0.12, 0.28] | 0.21 [0.12, 0.30] | 0.20 [0.12, 0.28] | 0.22 [0.13, 0.31] |
| **Age group** | | | | |
| 3-4 years | Reference | Reference | Reference | Reference |
| 4-5 years | -0.02 [-0.27, 0.22] | 0.02 [-0.24, 0.29] | -0.03 [-0.27, 0.21] | 0.02 [-0.24, 0.28] |
| 5-7 years | -0.09 [-0.36, 0.17] | -0.02 [-0.32, 0.27] | -0.09 [-0.35, 0.18] | -0.02 [-0.31, 0.27] |
| 7-9 years | 0.21 [-0.06, 0.47] | 0.25 [-0.05, 0.55] | 0.21 [-0.06, 0.47] | 0.24 [-0.05, 0.54] |
| **Sex** | | | | |
| Male | Reference | Reference | Reference | Reference |
| Female | 0.05 [-0.13, 0.23] | -0.01 [-0.21, 0.19] | 0.06 [-0.12, 0.23] | 0.00 [-0.20, 0.20] |
| **Indigenous identification** | | | | |
| Aboriginal | Reference | Reference | Reference | Reference |
| Torres Strait Islander | -0.12 [-0.52, 0.28] | -0.12 [-0.60, 0.37] | -0.14 [-0.53, 0.26] | -0.12 [-0.60, 0.35] |
| Both | -0.19 [-0.62, 0.24] | -0.30 [-0.79, 0.20] | -0.2 [-0.62, 0.22] | -0.34 [-0.83, 0.15] |
| **Diabetes status during pregnancy** | | | | |
| No diabetes |  | Reference |  | Reference |
| Diabetes |  | 0.24 [-0.16, 0.65] |  | 0.23 [-0.17, 0.63] |
| **Smoking during pregnancy** | | | | |
| No |  | Reference |  | Reference |
| Yes |  | 0.24 [0.04, 0.44] |  | 0.25 [0.05, 0.45] |
| **Weight gain during pregnancy** | | | | |
| Okay or not enough |  | Reference |  | Reference |
| Too much |  | 0.21 [-0.09, 0.51] |  | 0.18 [-0.12, 0.48] |
| **Area-level advantage/disadvantage at Wave 1** | | | | |
| Most advantaged |  |  | 0.01 [-0.26, 0.27] | -0.09 [-0.36, 0.19] |
| Mid-advantaged |  |  | Reference | Reference |
| Most disadvantaged |  |  | -0.50 [-0.83, -0.18] | -0.61 [-0.97, -0.26] |
| *n* | 861 | 682 | 861 | 682 |
| *ICC* | 0.07 | 0.06 | 0.05 | 0.04 |

* Each model includes all children with non-missing data on the included exposures for that model; thus, the number (n) varies between models with different exposures included. **Model 1**: adjusted for age group, sex and Indigenous identification. **^†^Model 2**: adjusted for variables in model 1 plus maternal diabetes, smoking, and weight gain during pregnancy. **^‡^Model 3**: adjusted for variables in model 1 plus area-level socioeconomic status. **^§^Model 4**: includes all variables.
