# Supplementary material for: Early Life Predictors of Increased Body Mass Index among Indigenous Australian Children
Source: PLoS One. 2015 Jun 15;10(6):e0130039. doi: 10.1371/journal.pone.0130039 (PMC4468174; doi:10.1371/journal.pone.0130039)
Supplement: S2 Table — (DOCX) [file pone.0130039.s003.docx]

**S2 Table.** **Distribution of birthweight among children providing data on BMI z-score in Wave 4 of LSIC (2011), across demographic and physiological variables.**

|  | **n** | **Mean birthweight z-score** | **95% CI** | **% SGA** | **% AGA** | **% LGA** |
| --- | --- | --- | --- | --- | --- | --- |
| **Total** | 861 | -0.17 | [-0.25, -0.10] | 16.5 | 72.9 | 10.6 |
|  |  |  |  |  |  |  |
| **Age** | | | | | | |
| 3-4 years | 200 | -0.06 | [-0.20, 0.08] | 10.5 | 78.0 | 11.5 |
| 4-5 years | 286 | -0.21 | [-0.33, -0.08] | 14.7 | 74.5 | 10.8 |
| 5-7 years | 187 | -0.08 | [-0.25, 0.09] | 18.2 | 70.6 | 11.2 |
| 7-9 years | 188 | -0.32 | [-0.50, -0.15] | 23.9 | 67.6 | 8.5 |
|  |  |  |  |  |  |  |
| **Sex** | | | | | | |
| Male | 433 | -0.13 | [-0.23, -0.02] | 16.9 | 71.6 | 11.6 |
| Female | 428 | -0.21 | [-0.32, -0.11] | 16.1 | 74.3 | 9.6 |
|  | | | | | | |
| **Indigenous identification** | | | | | | |
| Aboriginal | 759 | -0.19 | [-0.26, -0.11] | 16.7 | 73.0 | 10.3 |
| Torres Strait Islander | 60 | -0.03 | [-0.34, 0.28] | 16.7 | 66.7 | 16.7 |
| Both | 42 | -0.10 | [-0.45, 0.25] | 11.9 | 81.0 | 7.1 |
|  |  |  |  |  |  |  |
| **Maternal diabetes** | | | | | | |
| No diabetes | 799 | -0.19 | [-0.27, -0.11] | 17.0 | 72.6 | 10.4 |
| Yes diabetes | 56 | 0.22 | [-0.06, 0.49] | 5.4 | 80.4 | 14.3 |
| Missing | 6 | -1.02 | [-1.84, -0.19] | 50.0 | 50.0 | 0.0 |
|  |  |  |  |  |  |  |
| **Maternal smoking** | | | | | | |
| No smoke | 420 | 0.07 | [-0.04, 0.17] | 10.5 | 75.7 | 13.8 |
| Yes smoke | 402 | -0.39 | [-0.50, -0.28] | 22.1 | 70.2 | 7.7 |
| Missing | 39 | -0.43 | [-0.75, -0.10] | 23.1 | 71.8 | 5.1 |
|  |  |  |  |  |  |  |
| **Maternal weight gain** | | | | | | |
| Okay or not enough | 616 | -0.27 | [-0.36, -0.18] | 18.2 | 73.1 | 8.8 |
| Too much | 91 | 0.31 | [0.07, 0.56] | 9.9 | 71.4 | 18.7 |
| Missing | 154 | -0.06 | [-0.24, 0.13] | 13.6 | 73.4 | 13.0 |
|  |  |  |  |  |  |  |
| **Area-level advantage/disadvantage at Wave 1** | | | | | | |
| Most advantaged | 187 | -0.09 | [-0.24, 0.07] | 12.3 | 76.5 | 11.2 |
| Mid-advantaged | 564 | -0.21 | [-0.30, -0.11] | 17.6 | 72.5 | 9.9 |
| Most disadvantaged | 110 | -0.13 | [-0.37, 0.11] | 18.2 | 69.1 | 12.7 |

* Includes only the sample with no missing data on birthweight or BMI z-score. Size for gestational age categories were defined using cut-off points of z = -1.28 and z = +1.28 were used, in alignment with standard percentile cut-offs.
